# Occupational survey of the educational outputs of the first established program of cardiac technology speciality in the Kingdom of Saudi Arabia (2013–2022): A cross-sectional study

**DOI:** 10.1371/journal.pone.0295655

**Published:** 2023-12-14

**Authors:** Lamia Al Saikhan, Lamis Al Ghamdi

**Affiliations:** Department of Cardiac Technology, College of Applied Medial Sciences, Imam Abdulrahman Bin Faisal University, Dammam, Kingdom of Saudi Arabia; Tehran University of Medical Sciences, ISLAMIC REPUBLIC OF IRAN

## Abstract

**Background:**

The demand for qualified cardiac technology (CT) graduates is increasing in Saudi Arabia. This study aimed to provide the first descriptive occupational survey of the educational outputs of the first established CT speciality program in the Kingdom of Saudi Arabia over the past 10 years.

**Methods:**

This was a cross-sectional, survey-based study. An online self-administered survey was distributed to all alumni who graduated from the CT program between 2013 and 2022 at the Imam Abdulrahman bin Faisal University (IAU) in the Eastern Province of Saudi Arabia.

**Results:**

Of the 238 graduates, 164 completed the survey (72.2%). All the respondents (100%) were women, 56% were aged 25–30 years, 58.5% were married, the majority (95.7%) held a bachelor’s degree, and (93.3%) lived in the eastern region. Of the respondents, 33.7% specialised in cardiac catheterisation and 66.3% in echocardiography. Of those, 66.4% were employed, which was similar between both subspecialties and predominantly in the Eastern region (83.9%). A total of 57.9% of participants attributed the reason for unemployment to limited opportunities in the Eastern province (vs. 15.8% across the country). A total of 76.6% of the respondents reported that most job opportunities were in the Central region (Riyadh). The main barriers and limitations to unemployment reported by the alumni were the need for childcare assistance, further training, and poor job search/interview skills. Of the respondents, 42% expressed a desire to change their career path because of limited job opportunities (10.7%) followed by a change in career interest post-graduation (7.9%).

**Conclusions:**

The employment rate of IAU CT alumni was high (66.4%) and predominantly based in the Eastern region of Saudi Arabia, and 42% expressed a desire to change their career path due to limited regional job opportunities. The findings from this study will help inform the future of speciality across the kingdom and shape the potential for expansion.

## Introduction

Cardiovascular disease remains among the top-ranked causes of death in Saudi Arabia, although death due to specific non-communicable diseases, including cardiovascular disease, diabetes, and kidney disease, has decreased between 2010 and 2017 [1–3]. Cardiovascular risk factors, including obesity, smoking, high fasting plasma glucose concentration, and poor dietary habits, are considered major contributors to the disability burden among the Saudi Arabian population, as indicated by the results of the Saudi Health Interview Survey [1, 4–9]. As a result, the demand for healthcare services, particularly access to specialised cardiac care services, is expected to increase steadily as more than half of the Saudi Arabian population is below the age of 35 and the total population is expected to increase from 33.4 million in 2018 to 39.4 million in 2030 [10].

In an ideal cardiac care service department, the clinical care team typically consists of consultant cardiologists, cardiac nurses, cardiac technicians, and highly specialised cardiac technologists. The latter are typically graduates of a cardiac technology (CT) educational program, which remained several years unavailable in all academic institutions across the Kingdom of Saudi Arabia, with most having diplomas obtained in training-based programs. CT is an allied healthcare area focused on the diagnosis and management of patients with cardiovascular disease [11–13]. CT specialists (also called cardiac or cardiovascular technologists) are highly skilled professionals qualified to perform a range of cardiovascular investigations, improving the quality of patient care provided either by performing echocardiography (non-invasive), cardiac ultrasound examination, or by assisting with cardiac catheterisation procedures (invasive). In the United States, the overall employment of cardiac (cardiovascular) technologists is projected to grow by 10% from 2021 to 2031, faster than the average for all occupations [12].

As part of Vision 2030, improving the quality and efficiency of healthcare services is among the major objectives of the transformation program of the healthcare system announced by the Saudi Ministry of Health, focusing on improving preventive and therapeutic healthcare services [10, 14]. It is expected that the demand for CT graduates will increase across the Kingdom for the aforementioned reasons. In 2008/2009, the first CT program in the Kingdom of Saudi Arabia was established at Imam Abdulrahman bin Fasil University to overcome the limitation of a lack of skilled/qualified cardiac sonographers and specialists in cardiovascular invasive procedures [15]. The educational outputs of the program are closely related to the skills and professional expertise required to meet health needs across the country, which are in alignment with one of the strategic cornerstones by 2030: provide sufficient and capable health workforce [10, 14, 15]. The aim was to ensure the alignment of educational outputs with labour market needs.

Hence, this study aimed to provide the first descriptive occupational survey of the educational outputs of the first CT speciality program established in the Kingdom of Saudi Arabia over the past 10 years, including employment rate, regional distribution, barriers and limitations of unemployment, and potential for changing career paths. The findings from this study will help to plan the future of this speciality across the kingdom and shape the potential for expansion.

## Methods

### Study design and population

This was a cross-sectional survey-based study. An online self-administered survey was distributed to all alumni who graduated from the CT program at Imam Abdulrahman bin Faisal University (IAU) located in the Eastern Province (Dammam City) of Saudi Arabia between 2013 and 2022. Survey sampling was drawn from a list of all alumni, in which the contact information of all eligible participants was available. All potential eligible participants in the study were females since the program was only opened to female students. The survey was administered via Google Forms between 8^th^ August and 17^th^ November 2022, and voluntary and anonymous participation was sought to complete the online survey with up to four reminders sent to non-responders. No identifiable information was captured or requested from all participants.

The invitation letter included a message describing the purpose of the survey, clarifying the anonymity and confidentiality of the study, and providing the principal investigator with contact details. The study was approved by Imam Abdulrahman bin Faisal University Research Ethics Committee (IRB-2022-03-234, approval date: 15/06/2022), and was performed in accordance with the principles of the Helsinki Declaration. The collection and analysis method complied with the terms and conditions for the source of the data, and all participants provided informed written consent before participating in the study, which was assured by selecting ‘I confirm that I have read the section above and I understand that my participation is voluntary. I voluntarily agree to participate in the study before beginning the survey’. All relevant data can be found in this paper and the additional files.

### Instrument

The survey was distributed in English, as it is the official language of communication among healthcare workers in Saudi Arabia, and was developed based on current emerging evidence. The survey was designed to contain a structured multiple-choice response divided into four separate sections as follows: 1) demographic characteristics of the respondents (including age, gender, marital status, and area of living), 2) education/qualification (including year of graduation, subspeciality, and highest qualification), 3) employment-related factors (including barriers and limitations of unemployment, potential for pursuing a higher degree, and changing career path) and profession characteristics (including employment status, year of employment, job-related aspects [job sector, rank, title, salary, location of the job, and years of experience]), and 4) job satisfaction of the respondents based on responses on various statements recorded on a 6-point Likert scale. The questions in the ‘job satisfaction’ section were adapted from a previously developed and validated tool for measuring job satisfaction and commitment to work among healthcare workers [16, 17].

The first draft of the survey was evaluated for content and face validity and revised accordingly before being formally distributed among potential respondents. The survey contents were evaluated by two experts and amended as required. Regarding survey clarity and ambiguity, face validity was assessed by a survey pilot with ten participants. The final version of the survey was approved by the research team after considering the feedback and comments of the participants.

### Statistical analysis

Data analysis was performed using STATA 15.1 (StataCorp LLC, USA). Categorical variables were presented as counts (percentages). Differences between groups were assessed using χ^2^ test, Fisher exact test or proportion test, as appropriate. Statistical significance was defined as a two-tailed *P*-value <0.05. The required sample size was calculated based on available data using an online sample calculator (http://www.raosoft.com/samplesize.html; Raosoft Inc., Seattle, WA). The minimum sample size required for the survey was determined to be 139 participants, based on a chosen acceptable error margin of 5%, a 95% confidence level, and a 50% response distribution. In total, 238 graduates participated in the survey.

## Results

### Study population

Of the 238 CT graduates, 11 were lost to follow-up and excluded from the study. Of the remaining 227 participants, 164 (72.2%) completed the questionnaire. Table 1 summarises the demographic characteristics of the respondents. All respondents (100%) were women and 56% were aged 25–30 years. Of these, 58.5% were married, the majority (95.7%) held a bachelor’s degree, and (93.3%) lived in the eastern region. Of the respondents, 33.7% specialised in cardiac catheterisation (cath group) and 66.3% in echocardiography (echo group). Participants in the cath group were younger (<25 years, 56.4%, vs. 31.1%, *P* = 0.007) and had a higher tendency to live in other regions of Saudi Arabia (i.e., Western [3.6% vs. 0%] and Central [9.1% vs. 2.8%] regions, *P* = 0.052) than those in the echo group.

**Table 1:** Characteristics of the CT graduates (2012–2022).

Demographics, n (%)	All (n = 164)	Echo Group (n = 108)	Cath Group (n = 55)	P*
Age (years)				**0.007**
<25	65 (40.1)	33 (31.1)	31 (56.4)	
25–30	91 (56.2)	68 (64.2)	23 (41.8)	
>30	6 (3.7)	5 (4.7)	1 (1.8)	
Married	96 (58.5)	67 (62.0)	29 (52.7)	0.253
Have children	62 (37.8)	51 (47.2)	11 (20.0)	**0.001**
Area of living				0.052
Eastern region	153 (93.3)	104 (96.3)	48 (87.3)	
Northern region	0 (0.0)	0 (0.0)	0 (0.0)	
Western region	2 (1.2)	0 (0.0)	2 (3.6)	
Southern region	0 (0.0)	0 (0.0)	0 (0.0)	
Central region	8 (4.9)	3 (2.8)	5 (9.1)	
Other	1 (0.6)	1 (0.9)	0 (0.0)	
**Education, n (%)**				
Year of graduation				0.080
2012	1 (0.6)	1 (0.9)	0 (0.0)	
2013	5 (3.1)	4 (3.7)	1 (1.8)	
2014	7 (4.3)	7 (6.5)	0 (0.0)	
2015	19 (11.6)	15 (13.9)	4 (7.3)	
2016	13 (7.9)	9 (8.3)	4 (7.3)	
2017	13 (7.9)	8 (7.4)	5 (9.1)	
2018	9 (5.5)	4 (3.7)	5 (9.1)	
2019	30 (18.3)	25 (23.2)	5 (9.1)	
2020	19 (11.6)	11 (10.2)	7 (12.7)	
2021	18 (11)	8 (7.4)	10 (18.2)	
2022	24 (14.6)	11 (10.2)	13 (23.6)	
Subspeciality				
Cardiac Catheterizsation	55 (33.7)	-	-	
Echocardiography	108 (66.3)	-	-	
Highest qualification				0.510
Bachelor’s degree	157 (95.7)	102 (94.4)	54 (98.2)	
Master’s degree	6 (3.7)	5 (4.6)	1 (1.8)	
Doctorate degree (PhD, DPhil)	1 (0.61)	1 (0.9)	0 (0.0)	

Cath, cardiac catheterisation; Echo, echocardiography. *Comparing the Cath group vs the Echo group.

### Employment status of the CT graduates

Table 2 summarises the employment status of the CT graduates. The majority (97.1%) of CT graduates were predominantly classified by the Saudi Commission for Health Specialties (SCFHS) as specialists (98.5%), with no difference between the two subspecialties (cardiac catheterisation vs. echocardiography). Only 4.3% reported facing problems with the SCFHS classification, mainly due to difficulties with renewal after abruption from work. Of the 140 CT graduates, 66.4% were employed with no significant differences between the two subspecialties. Most of the employed alumni were based in the eastern region (n = 78 (83.9%)) followed by the Central (n = 8 (8.6%)) and Western regions (n = 7 (8.6%)).

**Table 2:** Employment status of the CT graduates.

n (%)	All (n = 140)	Echo (n = 97)	Cath (n = 42)	*P* *
Classified by SCFHS	136 (97.1)	94 (96.9)	41 (97.6)	0.818
*Classification*				0.358
Specialist	135 (98.5)	94 (97.9)	40 (100)	
Senior specialist	2 (1.5)	2 (2.1)	0 (0.0)	
Consultant specialist	0 (0.0)	0 (0.0)	0 (0.0)	
Problems with SCFHS	6 (4.3)	4 (4.2)	2 (4.8)	0.875
Currently employed	93 (66.4)	64 (66.0)	28 (66.7)	0.937
Reason for unemployment**				0.220
No opportunities across the country	3 (7.8)	2 (7.4)	1 (9.1)	
Limited opportunities across the country	6 (15.8)	2 (7.4)	4 (36.4)	
No opportunities in the Eastern province	7 (18.4)	5 (18.5)	2 (18.2)	
Limited opportunities in the Eastern province	22 (57.9)	18 (66.7)	4 (36.4)	
Location of most job opportunities from alumni experience				0.432
Eastern region	12 (8.8)	9 (9.6)	3 (7.1)	
Northern region	0 (0.0)	0 (0.0)	0 (0.0)	
Western (Mecca/Jeddah) region	20 (14.6)	16 (17.0)	4 (9.5)	
Southern (Asir) region	0 (0.0)	0 (0.0)	0 (0.0)	
Central (Riyadh) region	105 (76.6)	69 (73.4)	35 (83.3)	
Thought of changing career path	58 (42.0)	38 (39.6)	20 (48.8)	0.318

Ten subgroups were included in this analysis, as the last was from participants who were still interns (i.e., not yet graduated) at the time of the survey and, therefore, not eligible to apply for jobs.

*Comparing the Cath group vs the Echo group.

**n = 38. Abbreviation: SCFHS, Saudi Commission for Health Specialties.

The majority (57.9%) of CT graduates attributed the reason for unemployment to limited opportunities in the Eastern Province (vs. no opportunities, 18.4%), and only 15.8% attributed the reason for unemployment to limited opportunities across the country (vs. no opportunities, 7.8%), with no difference between the two subspecialities. Among the CT graduates, 76.6% reported that most job opportunities were in the central (Riyadh) region, with no difference between the two subspecialties. The proportions (95% confidence interval) of the four reasons for unemployment in the entire cohort were 0.157 (0.101–0.223 for limited opportunities in the Eastern Province, 0.05 (0.203–0.100) for no opportunities in the Eastern Province, 0.042 (0.015–0.090) for limited opportunities across the country, and 0.021 (0.004–0.061) for no opportunities across the country. Compared to other reasons of unemployment, limited opportunities in the Eastern Province appeared likely to be genuinely more important (p = 0.003 vs. no opportunities in the Eastern Province; p = 0.001 vs. limited opportunities across the country; and p<0.0001 vs. no opportunities across the country).

**Fig 1:**
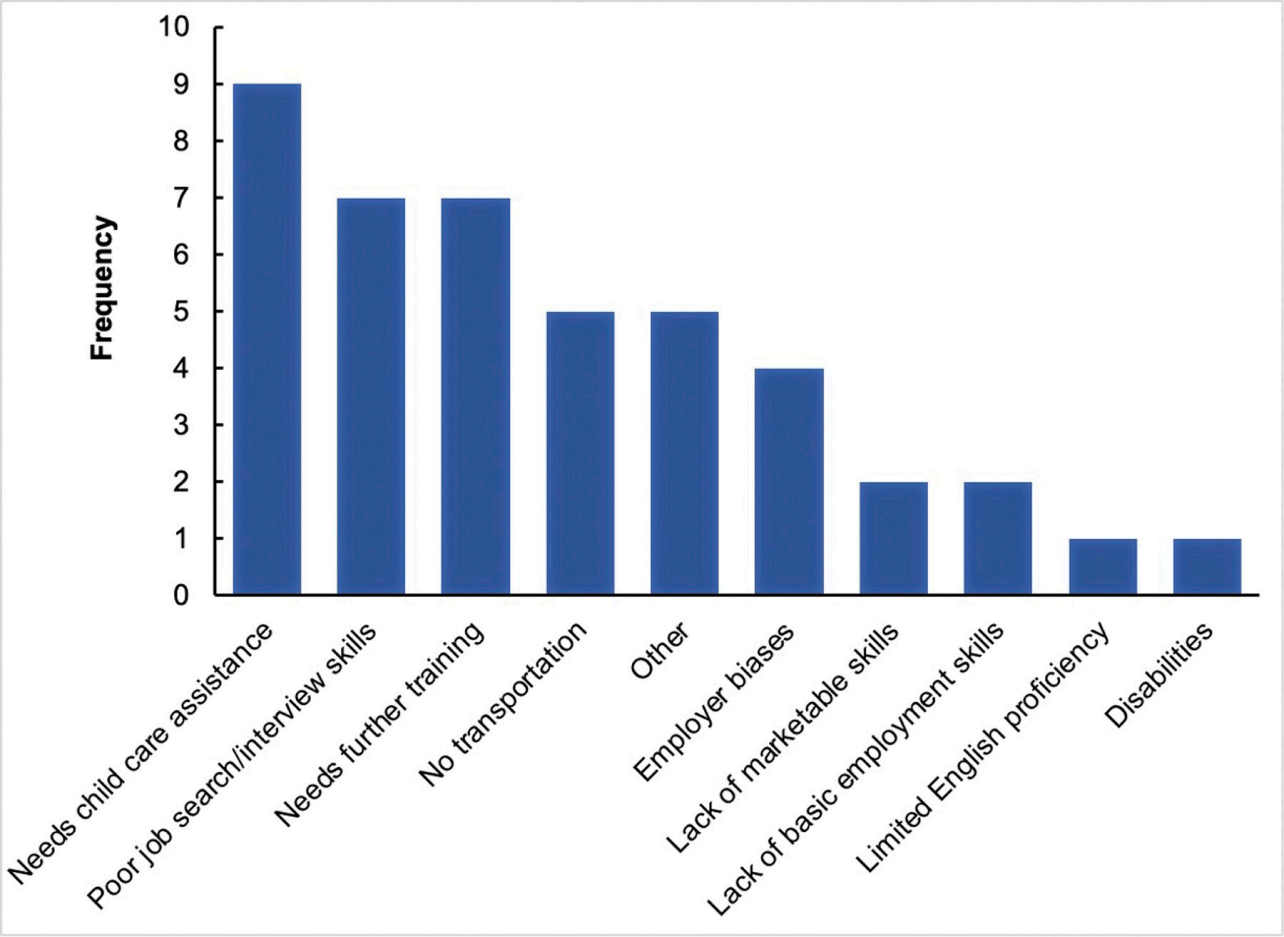
Barriers and limitations of unemployment.

**Fig 2:**
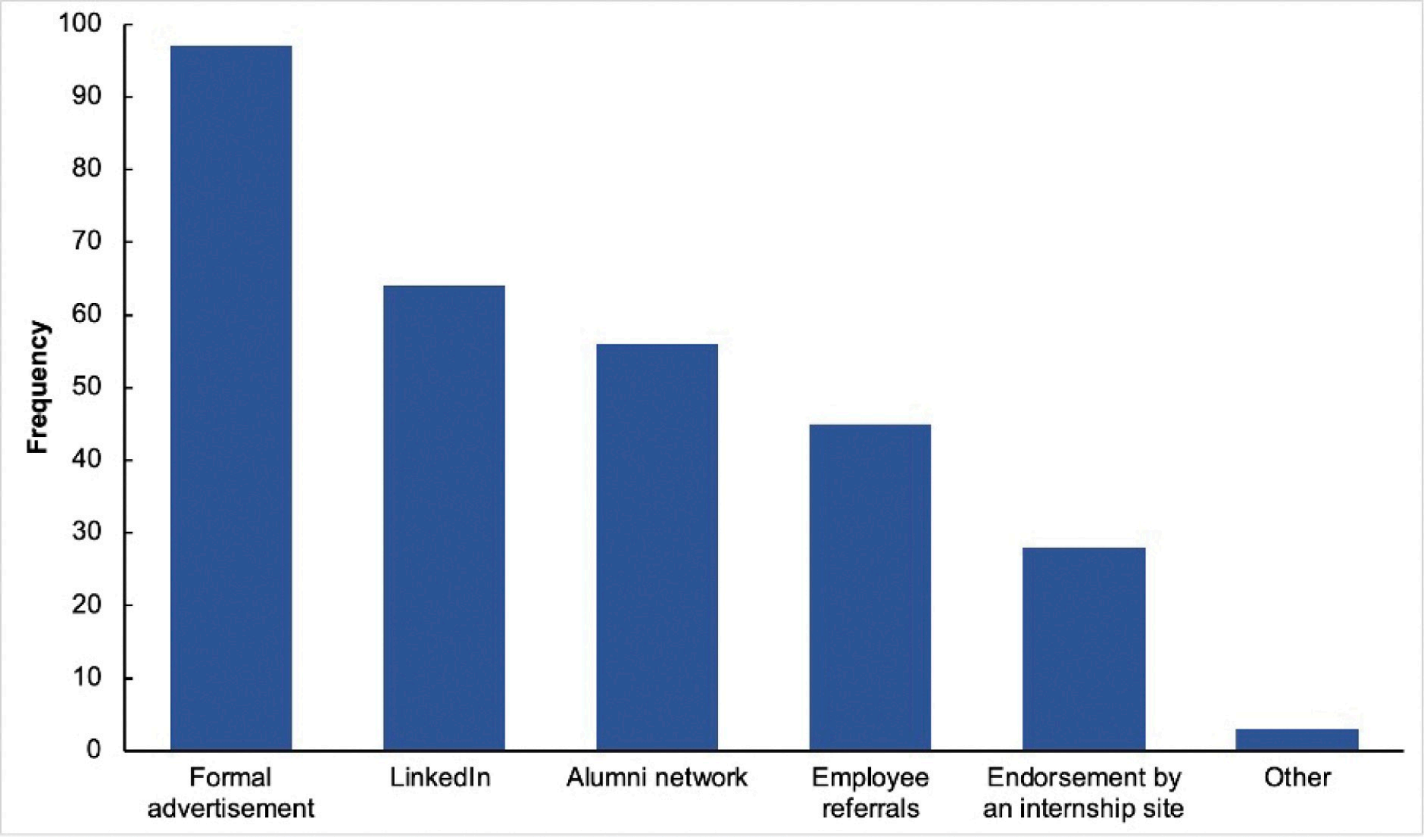
Common channels used by CT graduates for finding jobs.

The barriers and limitations to unemployment reported among CT graduates were mainly the need for childcare assistance (n = 9), poor job search/interview skills (n = 7), and the need for further training (n = 7), followed by a lack of transportation (n = 5) (Fig 1). Common channels used by CT graduates to find jobs were formal advertisement (n = 97), LinkedIn (n = 64), and alumni networks (n = 56), followed by employee referrals (n = 45) and endorsement by an internship site (n = 28) (Fig 2). Of the respondents, 42% expressed a desire to change their career paths, with no difference between the two subspecialties. The main reasons for this were limited regional job opportunities (10.7%) followed by a change in career preference post-graduation (7.9%) (see S1 Table).

### Employed CT graduates

Table 3 summarises the job characteristics of currently employed CT graduates. Of the respondents, 83.9% worked in the eastern region and 56.6% worked at government/public hospitals, with no significant difference between the subspecialties. The average working hours of the majority of respondents were 8–9 hours daily, equating to full-time employment status. Most participants had <1 year to 1–3 years of experience and a monthly income of SR 6, 001–10, 000 or SR 10, 001–15, 000, with no difference between the two subspecialties. Job rank according to the SCFHS aligned with the classification of the respondents at 85.9%, with no difference between the two subspecialties. Of the respondents, 26.4% reported that their work included day/night shift systems that were similar in both subspecialties. However, compared with the participants in the echo group, most of the work for those in the cath group included an overnight call system (70.4% vs. 39.7%, *P* = 0.002). The S2 Table summarises the previous job characteristics of currently employed CT graduates. The main reasons why CT graduates left their jobs were finding better offers (36%), salaries (20%), and working hours (18%) (see S3 Table). The job titles of CT graduates working in Saudi Arabia are shown in S4 Table. The medical institutes, hospitals, organisations, and workplaces that hired IAU CT graduates are shown in S5 Table.

**Table 3:** Job characteristics of currently employed CT graduates (n = 93).

n (%)	All (n = 93)	Echo (n = 64)	Cath (n = 28)	*P* *
Location of the medical institute/hospital/organization/workplace				0.119
Eastern region	78 (83.9)	56 (87.5)	21 (75.0)	
Northern region	0 (0.0)	0 (0.0)	0 (0.0)	
Western region	7 (8.6)	3 (4.7)	5 (17.9)	
Southern region	0 (0.0)	0 (0.0)	0 (0.0)	
Central region	8(8.6)	5 (7.8)	2 (7.1)	
Years of experience in the current job, (years)				0.527
<1	25 (26.9)	15 (23.4)	9 (26.1)	
1–3	40 (43.0)	28 (43.8)	12 (42.9)	
>3–5	15 (16.3)	13 (20.3)	2 (7.1)	
>5–7	11 (11.8)	7 (10.9)	4 (14.3)	
>7	2 (2.2)	1 (1.6)	1 (3.6)	
Job sector				0.974
Governmental/public hospital	51 (56.0)	36 (57.1)	15 (55.6)	
Private hospital	30 (32.9)	20 (31.8)	9 (33.3)	
Private clinic	3 (3.3)	2 (3.2)	1 (3.7)	
Industry	1 (1.1)	1 (1.6)	0 (0.0)	
Academia/university	6 (6.6)	4 (6.4)	2 (7.4)	
Overnight call system	44 (48.4)	25 (39.7)	19 (70.4)	**0.002**
Day/night shift system	24 (26.4)	17 (27.0)	7 (25.9)	0.741
Job rank according to the SCFHS				0.296
Technician	4 (4.4)	4 (6.3)	0 (0.0)	
Specialist	79 (85.9)	55 (85.9)	23 (85.2)	
Senior specialist	1 (1.1)	1 (1.6)	0 (0.0)	
Consultant specialist	0 (0.0)	0 (0.0)	0 (0.0)	
Not applicable	8 (8.7)	4 (6.3)	4 (14.8)	
Average salary				0.121
≤6000 SR	11 (12.0)	11 (17.5)	0 (0.0)	
6001–10,000 SR	36 (39.1)	22 (34.9)	13 (46.4)	
10,001–15,000 SR	40 (43.5)	27 (42.9)	13 (46.4)	
≥15,001 SR	5 (5.4)	3 (4.8)	2 (7.1)	
Total number of working hours per day				0.891
7	3 (3.2)	2 (3.1)	1 (3.6)	
8	41 (44.1)	27 (42.2)	13 (46.4)	
9	45 (48.4)	33 (51.6)	12 (42.9)	
10	2 (2.1)	1 (1.6)	1 (3.6)	
Other	2 (2.1)	1 (1.6)	1 (3.6)	
My postgraduate degree positively impacted my job	14 (15.7)	10 (16.7)	3 (10.7)	0.716

*Comparing the Cath group vs the Echo group.

## Discussion

This study provides the first descriptive occupational survey of the educational output of the first established CT speciality program in the Kingdom of Saudi Arabia over the past 10 years. Of the IAU CT alumni women included in this study, 66.4% were employed, which was similar between the echocardiography and cardiac catheterisation subspecialities. Most employed alumni were based in the Eastern region, justified by the location where the program was offered, followed by the Central and Western regions. The major reason for unemployment (from the perspective of the IAU CT alumni) was attributed to limited opportunities in the Eastern region, and most alumni believed that most job opportunities were in the Central (Riyadh) region. The main barriers and limitations to unemployment reported by the alumni were the need for childcare assistance, further training, and poor job search/interview skills. Nevertheless, 42% of the IAU CT alumni expressed a desire to change their career path due to limited job opportunities regionally, followed by a change in career interests post-graduation. The data reported in this study will be of great value to the Ministry of Health and/or the Ministry of Education to gain a clearer understanding of one of the major healthcare professions required to deliver a modern care model in Saudi Arabia [10].

CT is an internationally recognised allied health profession in which graduates from programs offering bachelor’s degrees are considered highly skilled and knowledgeable professionals qualified to perform a range of cardiovascular investigations and procedures [11–13], including echocardiography (non-invasive), cardiac ultrasound examination, and cardiac catheterisation (invasive). In contrast, cardiovascular technicians have less specialised job duties and tend to specialise mostly in the use of medical equipment to receive correct test results from patients, in addition to gaining most of their knowledge through on-the-job training with employers. The IAU, located in the Eastern Province (Dammam city) of Saudi Arabia, was the first university in the Middle East to offer a bachelor’s degree program in CT [15].

A part of the Saudi Health Sector Transformation Program is understanding the major specialties and subspecialties concerning regions and facilities for all categories of healthcare employees, including CT professionals [10]. This helps in forecasting the workforce demand and determining the gap between supply and demand annually. In this study, we provided the first descriptive occupational survey of the educational outputs of the first established CT speciality program in the Kingdom of Saudi Arabia over the past 10 years. All IAU CT alumni were women with an employment rate of 66.4%, similar between the two areas of specialities (echocardiography and cardiac catheterisation), and were predominantly based in the Eastern region, which could be justified by the location where the program is offered, followed by the Central and Western regions [15].

The major reason for unemployment (from the IAU CT alumni perspective) was limited opportunities in the Eastern region (only 15.8% attributed the reason for unemployment to limited opportunities across the country). Indeed, none of the IAU CT alumni worked in the Southern or Northern regions, and <10% worked in the Central and Western regions. Furthermore, most IAU CT alumni believed that most job opportunities were in the Central (Riyadh) region, the capital of Saudi Arabia. Furthermore, in this study, the main barriers and limitations of unemployment reported by the alumni were the need for childcare assistance, further training, and poor job search/interview skills. Despite the high employment rate of IAU CT alumni, 42% expressed a desire to change their career path due to limited job opportunities regionally, followed by a change in career interests post-graduation, in addition to developing work-related injuries.

Taken together, these data suggest that there is a need to draft a supply and demand strategy to increase and/or manage local supply, recruitment, and employee retention across the regions of Saudi Arabia. Most importantly, there is a need to coordinate with academic institutions, including IAU, to meet the market needs. For example, the potential to consider opening a section for men CT students, as they have a higher tendency to travel across the country after post-graduation for work outside their area of living compared to women for cultural reasons, will help in fulfilling the demands in regions other than the Eastern province. Furthermore, being women could also explain why there were only 33.7% of CT graduates specialising in cardiac catheterisation vs. 66.3% specialising in echocardiography, a non-invasive subspeciality that could suit Saudi women better than an invasive subspeciality [11, 13]. Cardiac catheterisation subspeciality, by contrast, includes overnight calls, weekend coverage and exposure to X-rays.

### Limitations

One limitation of this study is the potential bias due to non-response errors. This could be because some participants were unavailable, unable, or unwilling to participate. Consequently, we could not rule out potential differences between the excluded and included participants. Multiple notifications and follow-ups were attempted with nonrespondents at the implementation stage to maximise the response rate while minimising potential bias. Further, a limitation of this study is the relatively small sample size, and the study may lack external validity since the sample of the study included only cardiac technologist women working predominantly in Eastern Saudi Arabia. Therefore, it may not be possible to generalise our results to cardiac technologist men and cardiac technologists working in other parts of Saudi Arabia. Nevertheless, our study provides important preliminary data for guiding and informing future research work, including a more representative sample across all regions in Saudi Arabia.

In summary, although the employment rate of IAU CT alumni was high (66.4%), they were predominantly based in the Eastern region of Saudi Arabia, and 42% expressed a desire to change their career path due to limited job opportunities regionally, followed by a change in career interest post-graduation. The major reason for unemployment (from the IAU CT alumni perspective) was attributed to limited opportunities in the Eastern region, and the main barriers to unemployment reported by the IAU CT alumni were the need for childcare assistance, further training, and poor job search/interview skills. The findings from this study will help inform the future of speciality across the Kingdom of Saudi Arabia and shape the potential for expansion.

## Supporting information

S1 Table Reasons for changing career path (n = 140). (DOCX)

S2 Table Previous job characteristics of currently employed CT graduates (n = 50). (DOCX)

S3 Table Reasons for leaving previous first job post-graduation (n = 50). (DOCX)

S4 Table Summary of job titles for CT graduates working in Saudi Arabia. (DOCX)

S5 Table List of reported medical institutes, hospitals, organizations, or workplaces for currently employed cardiac technologists who graduated from IAU (n = 93). (DOCX)
