# Supplementary material for: Occupational survey of the educational outputs of the first established program of cardiac technology speciality in the Kingdom of Saudi Arabia (2013–2022): A cross-sectional study
Source: PLoS One. 2023 Dec 14;18(12):e0295655. doi: 10.1371/journal.pone.0295655 (PMC10721097; doi:10.1371/journal.pone.0295655)
Supplement: S1 Table — (DOCX) [file pone.0295655.s001.docx]

| **Table S1 Reasons for changing career path (n = 140)** | |
| --- | --- |
| Limited job opportunity | 15 (10.7) |
| Changed preference after graduation | 11 (7.9) |
| Work-related injury | 5 (3.6) |
| Absence of motive and promotions in the clinical field | 4 (2.9) |
| Salary | 3 (2.1) |
| Working hours | 3 (2.1) |
| Personal reasons | 2 (1.4) |
| No postgraduate studies opportunities in Saudi Arabia | 1 (0.7) |
| Issues with the job description for cardiac catheterization specialist | 1 (0.7) |
| Environment | 1 (0.7) |
