# Supplementary material for: Occupational survey of the educational outputs of the first established program of cardiac technology speciality in the Kingdom of Saudi Arabia (2013–2022): A cross-sectional study
Source: PLoS One. 2023 Dec 14;18(12):e0295655. doi: 10.1371/journal.pone.0295655 (PMC10721097; doi:10.1371/journal.pone.0295655)
Supplement: S2 Table — (DOCX) [file pone.0295655.s002.docx]

| **Table S2 Previous job characteristics of currently employed CT graduates (n = 50)** | | | | |
| --- | --- | --- | --- | --- |
| n (%) | **All** | **Echo** | **Cath** | ***P*** |
| Location of the medical institute/hospital/organization/workplace |  |  |  | **0.005** |
| Eastern region | 42 (84.0) | 34 (94.4) | 8 (57.1) |  |
| Northern region | 0 (0.0) | 0 (0.0) | 0 (0.0) |  |
| Western region | 1 (2.0) | 0 (0.0) | 1 (7.1) |  |
| Southern region | 0 (0.0) | 1 (2.6) | 0 (0.0) |  |
| Central region | 7 (14.0) | 2 (5.6) | 5 (35.7) |  |
| Years of experience, (years) |  |  |  | 0.878 |
| <1 | 26 (52.0) | 18 (50.0) | 8 (57.1) |  |
| 1 | 9 (18.0) | 6 (16.7) | 3 (21.4) |  |
| 2 | 11 (22.0) | 8 (22.2) | 3 (21.4) |  |
| 3 | 2 (4.0) | 2 (5.6) | 0 (0.0) |  |
| 4 | 1 (2.0) | 1 (2.8) | 0 (0.0) |  |
| 5 | 1 (2.0) | 1 (2.8) | 0 (0.0) |  |
| Job sector |  |  |  | 0.066 |
| Governmental/public hospital | 15 (30.0) | 8 (22.2) | 7 (50.0) |  |
| Private hospital | 28 (56.0) | 21 (58.3) | 7 (50.0) |  |
| Private clinic | 7 (14.0) | 7 (19.4) | 0 (0.0) |  |
| Overnight call system | 12 (24.5) | 3 (8.6) | 9 (64.3) | **<0.0001** |
| Day/night shift system | 17 (34.0) | 16 (44.4) | 1 (7.1) | **0.012** |
| Job rank according to the SCFHS |  |  |  | 0.675 |
| Technician | 5 (10.0) | 4 (11.1) | 1 (7.1) |  |
| Specialist | 45 (90.0) | 32 (88.9) | 13 (92.9) |  |
| Average salary |  |  |  | 0.093 |
| ≤6000 SR | 19 (38.0) | 17 (47.2) | 2 (14.3) |  |
| 6001–10,000 SR | 25 (50.0) | 15 (41.7) | 10 (71.4) |  |
| 10,001–15,000 SR | 6 (12.0) | 4 (11.1) | 2 (14.3) |  |
| Total number of working hours per day |  |  |  | 0.421 |
| 8 | 29 (58.0) | 22 (61.1) | 7 (50.0) |  |
| 9 | 19 (38.0) | 12 (33.3) | 7 (50.0) |  |
| 10 | 2 (4.0) | 2 (5.6) | 0 (0.0) |  |
| My postgraduate degree positively impacted my job | 2 (4.2) | 2 (5.9) | 0 (0.0) | 0.644 |
