# Supplementary material for: Occupational survey of the educational outputs of the first established program of cardiac technology speciality in the Kingdom of Saudi Arabia (2013–2022): A cross-sectional study
Source: PLoS One. 2023 Dec 14;18(12):e0295655. doi: 10.1371/journal.pone.0295655 (PMC10721097; doi:10.1371/journal.pone.0295655)
Supplement: S3 Table — (DOCX) [file pone.0295655.s003.docx]

| **Table S3 Reasons for leaving previous first job post-graduation (n = 50)** | |
| --- | --- |
| Finding better offer | 18 (36) |
| Salary | 10 (20) |
| Working hours | 9 (18) |
| Location | 4 (8) |
| Personal circumstances | 4 (8) |
| Working days | 3 (6) |
| The institute closed permanently | 3 (6) |
| Job responsibilities differ from subspeciality | 2 (4) |
| Toxic environment | 2 (4) |
| Management | 2 (4) |
| Work overload | 2 (4) |
| Temporary contract | 2 (4) |
| Transportation | 1 (2) |
| Lack of development opportunity | 1 (2) |
| Postgraduate education | 1 (2) |
