# Supplementary material for: Occupational survey of the educational outputs of the first established program of cardiac technology speciality in the Kingdom of Saudi Arabia (2013–2022): A cross-sectional study
Source: PLoS One. 2023 Dec 14;18(12):e0295655. doi: 10.1371/journal.pone.0295655 (PMC10721097; doi:10.1371/journal.pone.0295655)
Supplement: S4 Table — (DOCX) [file pone.0295655.s004.docx]

| **Table S4 Summary of job titles for CT graduates working in Saudi Arabia** | |
| --- | --- |
| **Based on the current job** | **[n = 93, n (%)]** |
| Cardiovascular technologist | 17 (18.3) |
| Supervisor - cardiovascular technologist | 2 (2.2) |
| Cardiovascular technology specialist | 4 (4.3) |
| Cardiovascular specialist | 3 (3.2) |
| Cardiac technologist | 15 (16.1) |
| Adult and paediatric cardiac technologists | 1 (1.1) |
| Cardiac technology specialist | 15 (16.1) |
| Cardiac technician | 1 (1.1) |
| Associate cardiovascular technician | 1 (1.1) |
| Echocardiography technologist | 7 (7.5) |
| Associate echocardiogram technologist | 1 (1.1) |
| Echocardiography specialist | 5 (5.4) |
| Cardiac sonographer | 1 (1.1) |
| Echo technician | 2 (2.2) |
| Cardiac catheterization technologist | 2 (2.2) |
| Invasive cardiac specialist | 1 (1.1) |
| Cath lab technologist | 3 (3.2) |
| Cath lab specialist | 1 (1.1) |
| Non-invasive cardiac technologist | 1 (1.1) |
| ECG specialist | 2 (2.2) |
| ECG technician | 1 (1.1) |
| Demonstrator – academia | 3 (3.2) |
| Assistant professor – academia | 1 (1.1) |
| **Based on the previous first job** | **[n = 50, n (%)]** |
| Cardiovascular technologist | 5 (10) |
| Cardiovascular specialist | 2 (4) |
| Cardiac technologist | 15 (30) |
| Cardiac technology specialist | 10 (20) |
| Cardiovascular technician | 1 (2) |
| Echocardiography technologist | 3 (6) |
| Echocardiography specialist | 4 (8) |
| Echocardiography technician | 1 (2) |
| Paediatric echocardiographer | 1 (2) |
| Cardiac catheterization technologist | 1 (2) |
| Cardiac rhythm management specialist | 1 (2) |
| Cath lab technician | 1 (2) |
| Non-invasive cardiovascular technologist | 1 (2) |
| ECG specialist | 1 (2) |
