# Supplementary material for: Occupational survey of the educational outputs of the first established program of cardiac technology speciality in the Kingdom of Saudi Arabia (2013–2022): A cross-sectional study
Source: PLoS One. 2023 Dec 14;18(12):e0295655. doi: 10.1371/journal.pone.0295655 (PMC10721097; doi:10.1371/journal.pone.0295655)
Supplement: S5 Table — (DOCX) [file pone.0295655.s005.docx]

| **Table S5 List of reported medical institutes, hospitals, organizations, or workplaces for currently employed cardiac technologists who graduated from IAU (n = 93)** | |
| --- | --- |
| **Eastern region** | n (%) |
| King Fahad University Hospital | 9 (9.7) |
| King Fahad Military Medical Complex | 6 (6.5) |
| National Guard Hospital | 2 (2.2) |
| Qatif Central Hospital | 3 (3.2) |
| King Fahad Specialist Hospital | 2 (2.2) |
| Dr. Suliman Al-Habib Medical Group | 4 (4.3) |
| Saudi German Hospital | 3 (3.2) |
| Saud Albabtain Cardiac Centre | 6 (6.5) |
| Maternity and Children Hospital | 2 (2.2) |
| Mouwasat Hospital | 5 (5.4) |
| Royal Commission Hospital | 3 (3.2) |
| Johns Hopkins Aramco Healthcare | 2 (2.2) |
| King Abdulaziz Medical City (Imam Abdulrahman Al Faisal Hospital – National Guard Hospital) | 3 (3.2) |
| Prince Sultan Cardiac Centre | 1 (1.1) |
| Dar Afiah Medical Centre | 1 (1.1) |
| Dr Alnaamy Specialist Hospital | 1 (1.1) |
| ProCare Hospital | 1 (1.1) |
| Arrawdha General Hospital | 1 (1.1) |
| Imam Abdulrahman bin Faisal University | 6 (6.5) |
| Almana General Hospital | 4 (4.3) |
| Alsalam Hospital | 1 (1.1) |
| Mohammad Aldossary Hospital | 1 (1.1) |
| **Central (Riyadh) region** | n (%) |
| King Saud Medical City | 2 (2.2) |
| King Fahad Cardiac Centre at King Khalid University Hospital | 1 (1.1) |
| King Faisal Specialist Hospital and Research Centre | 2 (2.2) |
| King Fahad Medical City | 1 (1.1) |
| **Western (Mecca/Jeddah) region** | n (%) |
| Madinah Cardiac Centre | 1 (1.1) |
| Royal Commission Medical Centre - Yanbu | 2 (2.2) |
| **Northern region** | n (%) |
| King Salman Specialist Hospital | 1 (1.1) |
| **Other** | n (%) |
| Mohammed Bin Khalifah Specialist Hospital | 1 (1.1) |
| Ministry of Health | 1 (1.1) |
